# Bone marrow endosteum in homeostasis and metastasis

**DOI:** 10.1098/rsob.250103

**Published:** 2025-10-01

**Authors:** Yuta Nakai, Wanida Ono, Noriaki Ono

**Affiliations:** ^1^ Department of Diagnostic and Biomedical Sciences, School of Dentistry, The University of Texas Health Science Center at Houston, Houston, TX, USA; ^2^ Division of Orthodontics, School of Dentistry, University of California, San Francisco, San Francisco, CA USA

**Keywords:** bone marrow, endosteum, skeletal stem cells, bone metastasis, stem cell niche, osteosarcoma

## Abstract

The endosteum is a thin layer of connective tissue lining the inner surfaces of bones adjoining the medullary cavity. The endosteum houses a variety of cells crucial for bone growth, repair and remodelling, including bone-forming osteoblasts, bone-resorbing osteoclasts and their precursor cells. Historically, the endosteum has been extensively studied as a key site for haematopoiesis by which blood cells are incessantly produced. However, recent studies have defined the endosteum as a niche for skeletal stem cells, underscoring the importance of the harmony between the inner endosteum and the outer periosteum in maintaining bone homeostasis. The endosteum also plays a significant role in pathological conditions, as it is recognized as a preferential site for bone metastasis of several common carcinomas, including breast and prostate cancers. The uniquely complex microenvironment of the endosteum favours the survival of cancer cells, contributing to dormancy, resistance to therapies and eventually, reemergence and progression. In this review, we discuss the multifaceted functions of the bone marrow endosteum, focusing on its dual roles in normal bone haematopoiesis and tumour metastasis.

**Figure:**
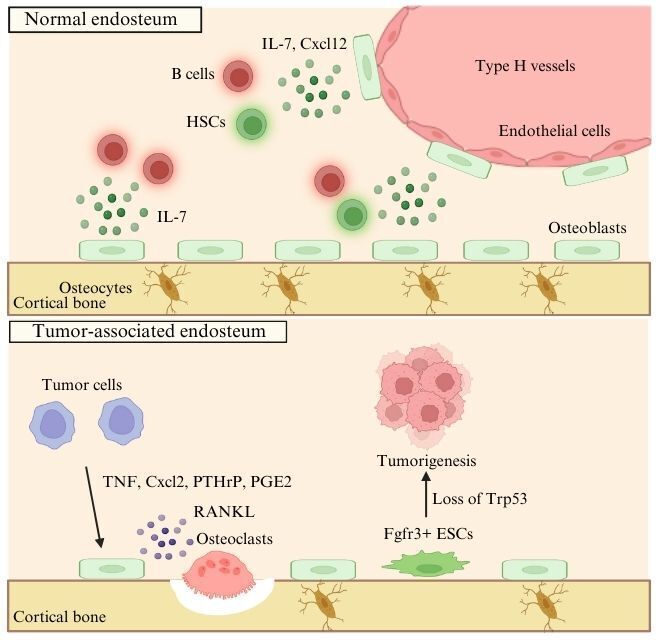

## 1. Introduction

The skeleton is maintained by diverse cell types of the mesenchymal cell lineage, including bone-forming osteoblasts, fat-forming adipocytes, cartilage-forming chondrocytes and their precursors of stromal cell populations, while housing cells of the haematopoietic lineage within its marrow cavity [1,2]. Beyond its central role in locomotion, the bone has also been recognized for its extensive functions, such as the site of haematopoiesis and the target of mineral homeostasis through endocrine regulations [1,3]. Osteoblasts, the only cells capable of depositing mineralized matrix, play a central role in supporting the skeleton. Osteoblasts need to be replenished by skeletal stem cells (SSCs) to keep building bones, as osteoblasts have only a limited lifespan owing to their specialized function in matrix production and mineralization and their eventual attrition from apoptosis or embedment as osteocytes [4].

SSCs were first discovered in the bone marrow stroma [5,6]. When isolated *ex vivo* and grown under cultured conditions, these cells can self-renew and differentiate into osteoblasts, chondrocytes and adipocytes. Fluorescence-activated cell sorting (FACS)-based isolation techniques using appropriate combinations of cell surface markers facilitated the characterization of SSCs [7]. In addition, *in vivo* fate-mapping approaches using cell type-specific constitutively active *cre* drivers, as well as *in vivo* lineage-tracing approaches using cell type-specific tamoxifen-inducible *creER* drivers in mice, have also been instrumental for elucidating *in vivo* functions of SSCs [7–12]. Accumulating evidence indicates that skeletal development, maintenance and regeneration are coordinated by diverse classes of SSCs localized in each compartment of the bone [8,9,13].

Long bones are shaped through two different mechanisms of endochondral and intramembranous bone formation. The growth plate is a unique cartilaginous structure located at the end of the endochondral bone. It is an essential component of endochondral bones, composed of three main layers: the resting, proliferating and hypertrophic zones [14]. In endochondral bone formation, longitudinal axial growth of long bones is driven at least in part by the proliferation and differentiation of growth plate stem cells (GPSCs) residing in the resting zone. Our group previously reported that parathyroid hormone-related protein (PTHrP)-expressing GPSCs are the source of growth plate chondrocytes, osteoblasts and bone marrow stromal cells [8]. PTHrP^+^ GPSCs first differentiate into proliferating and hypertrophic chondrocytes and, subsequently, into osteoblasts as well as marrow stromal cells beneath the growth plate, including C-X-C chemokine ligand (CXCL)12-abundant reticular (CAR) cells [8]. The current concept is that SSCs in the resting zone of the growth plate are pivotal to endochondral bone formation.

**Figure 1.**
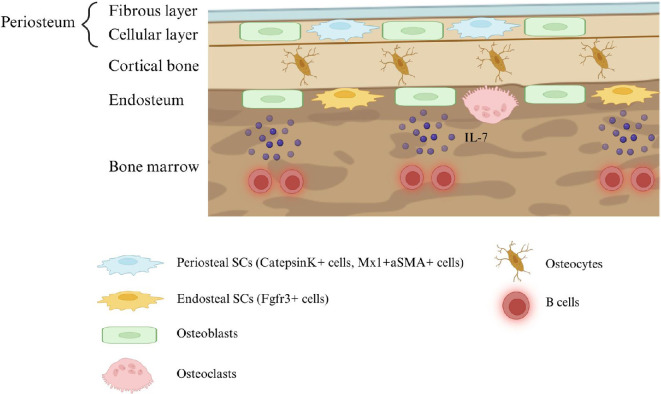
The schematic representation of the bone microenvironment. The figure illustrates the periosteum, cortical bone, endosteum and bone marrow. The periosteum comprises a fibrous layer and a cellular (cambium) layer containing periosteal stem cells (cathepsin K^+^ cells and Mx1^+^aSMA^+^ cells), contributing to bone regeneration. The endosteum, lining the inner surface of the cortical bone, harbours endosteal stem cells (Fgfr3^+^ cells) and plays a crucial role in bone remodelling through the activity of osteoblasts and osteoclasts. Osteoblasts in the endosteum constitute a niche for B cells through the production of IL-7.

In intramembrane bone formation, SSCs within the periosteum, termed periosteal stem cells (PSCs), coordinate the growth of long-tubular bone in the transverse axial direction [15,16]. Histologically, the periosteum is the site, where the skeletal muscle is attached, composed of two fibrous and cambium (cellular) layers [17,18] (figure 1). The outer fibrous layer is rich in collagen and elastin and includes linear neuron fibres. In contrast, the inner cambium layer comprises several types of cells, including osteoblast precursor cells and fibroblasts. The cambium layer is highly vascularized with a high density of microvessels [18,19]. Cathepsin K^+^ and Mx1^+^aSMA^+^ PSCs within the periosteal inner layer can self-renew and differentiate into tri-lineage cells in transplantation and promote osteogenesis [16,20]. In this process, PSCs directly supply osteoblasts without chondrogenic differentiation.

Relatively less defined are SSCs residing in the endosteum, the counterpart of the periosteum inside of the cortical bone (figure 1). The endosteum includes diverse cell types, including osteoblasts and their precursor cells and endothelial cells [7,9]. The endosteum is a thin layer lining the inner surface of the bone adjoining the bone marrow cavity. Historically, the endosteum has been primarily studied as a haematopoietic stem cell (HSC) niche component [3,21]. However, recent studies show that skeletal stem cells in the endosteum, termed endosteal stem cells (ESCs), coordinate active bone formation, particularly in young bones, while also being involved in initiating and progressing osteosarcoma-like lesions [9]. Therefore, understanding the characteristics of the endosteum is essential not only for understanding the fundamental mechanism of bone growth and development but also for exploiting a potential therapeutic target of pathological conditions in bone and blood, such as osteosarcoma, osteoporosis and multiple myeloma. This review summarizes the current understanding of the endosteum with an emphasis on the functional roles of endosteal cells in primary and metastatic tumours.

## 2. Bone marrow endosteum as a haematopoietic stem and progenitor cell niche

The endosteal and perisinusoidal niches are the two main HSC-supporting niches in the bone marrow microenvironment. The perisinusoidal niche, also called the central or perivascular niche, houses approximately 85% of the HSCs. In comparison, the endosteal niche at the interface between the bone and bone marrow houses the remaining 15% of HSCs [3,22]. The endosteal niche is also enriched with mesenchymal lineage cells, such as osteoblasts and osteoblast progenitor cells. Osteoblasts on the endosteal surface are integral to supporting haematopoietic progenitor cells, which are thought to play multifaceted roles in creating a haematopoiesis-supporting microenvironment, or niche, ensuring the proper regulation of HSC quiescence, self-renewal and differentiation [3,23]. The dynamic interplay between osteoblasts and haematopoietic progenitor cells is essential for sustaining a balanced production of blood cells throughout life [3,21,23–25]. Endosteal osteoblasts express the receptor for parathyroid hormone (PTH) and parathyroid hormone-related protein (PTHrP) (PTH1R). Mice expressing a constitutively active PTH1R under the control of the α1(I) collagen promoter active in osteoblastic cells (Col1a1-caPTH1R) show a dramatic increase in osteoblastic cells with an upregulation of a Notch ligand Jagged 1, which supports an increase of HSCs through Notch signalling activation [23]. Also, conditional deletion of bone morphogenic protein receptor type IA (BMPRIA) by *Mx1-cre* induces a drastic increase in osteoblasts and HSCs, supporting the notion that osteoblasts are a component of the HSC niche [24]. The converse experiment of ablating osteoblasts using Col2.3Δtk transgenic mice reduces the HSC number [26]. Other studies show that HSCs prefer to engraft at the endosteal region after transplantation [27,28]. On the other hand, several studies have shown that the osteoblast number is not correlated with the HSC number. Moreover, CXCL12 and stem cell factor (SCF), two cytokines critical for HSC maintenance, from the endosteum are dispensable for maintaining HSCs. Conditional deletion of CXCL12 in osteoblasts selectively impairs B cell development without affecting the HSC number [29]. Likewise, conditional deletion of SCF in osteoblasts does affect HSC maintenance [30]. Cecal ligation and puncture is a widely used model of polymicrobial sepsis. This triggers a systemic inflammatory response, leading to a cytokine storm. In addition to affecting immune cells, cecal ligation and puncture also impact bone marrow mesenchymal cells, leading to the loss of osteoblasts. Osteoblastic ablation induced by cecal ligation reduced the number of T and B lymphocytes and common lymphoid progenitor cells (CLPs) without affecting the HSC number [25]. This immunodeficient phenotype is rescued by intermittent PTH administration [31].

Therefore, the precise role that endosteal osteoblasts play in HSC maintenance remains largely unclear. However, multiple lines of evidence support that the endosteal niche is essential for maintaining their downstream haematopoietic progenitor cell populations, including lymphoid progenitor cells, supporting the concept that the endosteum is an essential haematopoiesis-supporting structure.

## 3. Bone marrow endosteum in haematological tumour progression

**Figure 2.**
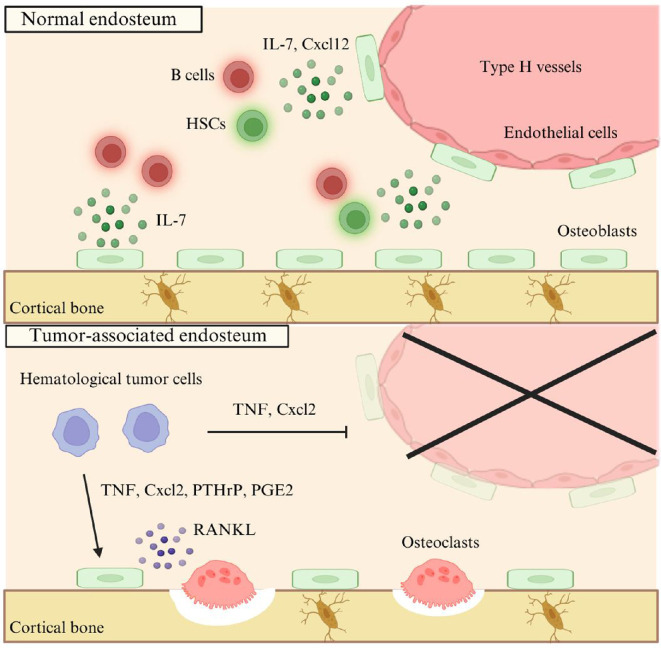
Normal and haematological tumour-affected endosteal microenvironment. The upper panel shows the normal endosteum. Type H vessels run through the endosteum and are crucial in maintaining osteoblasts. These osteoblasts and endothelial cells contribute to forming B cell and HSC niches by producing IL-7 and Cxcl12. The lower panel represents the haematological tumour-affected endosteum. Haematological tumour cells alter the bone microenvironment by secreting tumour necrosis factor (TNF) and Cxcl2. These factors impair the balance of bone remodelling by inducing RANKL, which promotes osteoclastogenesis and excessive bone resorption. Furthermore, tumour cells reduce osteoblasts by destroying Type H vessels through TNF and Cxcl2 production. This dysregulated interaction contributes to bone degradation and disease progression.

The emerging concept is that the endosteum can also serve as a niche for haematological tumours, including multiple myeloma (MM) and acute myeloid leukaemia (AML) (figure 2). MM is a malignant neoplasm characteriszd by the clonal proliferation of plasma cells within the bone marrow, producing abnormal monoclonal immunoglobulins or light chains [32]. This accumulation of malignant plasma cells disrupts normal bone marrow function, causing anaemia, immunodeficiency and renal dysfunction. One of the hallmark features of MM is the presence of osteolytic lesions, which result in significant morbidity for patients, including recurrent fractures [33]. Osteolytic lesions of MM show unique spot-like bone erosion in the cortical bone, called punched-out lesions [34], distinct from those developing from bone metastases of epithelial cell-derived tumours, such as breast cancer and lung cancer [33,35]. One reason for this unique osteolysis is that MM cells colonize the endosteum. Lawson *et al.* show that 5TGM1-GFP mouse-derived myeloma cells preferentially colonized endosteal bone surfaces by two-photon microscopy using 1,1'-dioctadecyl−3,3,3',3'-tetramethylindodicarbocyanine (DiD) labelling [36]. As 5TGM1-GFP DiD^hi^ cells divide, DiD becomes progressively diluted among their descendants. Dormant 5TGM1-GFP DiD^hi^ cells are preferentially localized to the endosteal surface, stay dormant over time and are resistant to chemotherapy. Interestingly, increased osteoclast activity can disrupt the endosteal niche, potentially leading to the reactivation of dormant MM cells and subsequent cortical bone erosion. Conversely, inhibiting osteoclast activity helps maintain the dormancy of MM cells [36].

AML is a heterogeneous and aggressive haematologic malignancy characterized by the rapid proliferation of abnormal myeloid precursors (blasts) in the bone marrow, blood and other tissues. The pathogenesis of AML involves a complex interplay of genetic, epigenetic and environmental factors that lead to the disruption of normal haematopoiesis and uncontrolled growth of leukaemic cells [37,38]. AML, like MM, is also known to grow and proliferate in the endosteum. AML cells preferentially colonize the osteoblast-rich areas, such as the trabecular bone and endosteum, altering the endosteal niche [39]. These cells compete with HSCs and occupy the endosteal niche, impairing normal haematopoiesis [40]. The *in vivo* AML mouse model shows that vasculature loss is observed in the endosteum but not in the central bone marrow [40]. This phenotype is also seen in over 80% of BM trephine biopsies from patients with AML [40]. AML cells inside the endosteum have a higher expression of genes related to inflammatory responses and anti-angiogenic cytokine *Cxcl2,* downstream of tumour necrosis factor (TNF) signalling pathways [40]. These cytokines are postulated to disturb the endosteal vasculature. The first-line treatment for AML is chemotherapy, such as cytarabine or anthracyclines. Chemotherapy reaches bone marrow through blood vessels but is ineffective in annihilating endosteum-residing AML cells due to disrupted endosteal vasculature [39,40]. In other words, AML cells may protect themselves from chemotherapy by destroying blood vessels within their own niche in the endosteum.

Clinically, AML is characterized by impaired osteogenesis and decreased osteocalcin levels in the peripheral blood [41]. In recent years, the critical roles that blood vessels play in bone biology have been unveiled [42]. Two distinct types of blood vessels, known as type H and type L vessels, have been identified as crucial players in bone homeostasis. Type H vessels are predominantly located in the metaphyseal regions of long bones and near the endosteal surface. In contrast, type L vessels are primarily found in the diaphyseal region of bones. Type H vessels are characterized by high expression of CD31 (also known as PECAM-1) and endomucin, which are markers of endothelial cells and are associated with areas of active bone formation by providing a niche that supports the proliferation and differentiation of osteoprogenitor cells [43]. During AML pathogenesis, several blood vessels, including CD31^hi^Endomucin^hi^ type H vessels, are impaired, associated with osteoblast dysfunction and the reduced number of HSCs [40,44,45]. The collapse of both the endosteal and perisinusoidal niches may cause decreased HSCs in AML. Overall, the endosteum provides a crucial niche for haematological tumour cells, where tumour cells preferentially remodel the microenvironment for their survival and progression (figure 2).

## 4. Bone marrow endosteum in bone metastasis

Bone is one of the most preferential metastatic sites of malignant tumours such as breast cancer, lung cancer and malignant melanoma [35]. When cancer cells migrate to the bone marrow, they establish a metastatic site within the bone microenvironment, which will eventually provide a seedling bed for cancer cells to grow and destroy bones, leading to significant morbidity associated with pain, fractures and hypercalcemia. Recent advances in cancer therapies have extended patients' longevity and, conversely, increased the risk of bone metastasis. Cancer cells originating in the primary site often metastasize to the bone through the bloodstream by chemotactic factors such as CXCL12 and receptor activator of nuclear factor-kB ligand (RANKL) [46,47]. The endosteum is rich in osteoblasts, often attracting tumour cells to the endosteum through RANKL [48]. Cancer cells metastasizing to bone compete with HSCs for niche occupancy and then acquire dormancy [49]. Wang *et al.* show that systemically inoculated tumour cells colonize near type I collagen (COL-I)^+^ or alkaline phosphatase (ALP)^+^ cells [50]. *in vitro* experiments further reveal that the cell–cell contact between tumour cells and osteoblasts accelerates tumour progression [50]. E-cadherin is expressed in several types of breast cancer cells and is negatively associated with overall survival and distant metastasis-free survival [51]; the interaction between E-cadherin on tumour cells and N-cadherin on osteoblasts accelerates tumour proliferation [50]. After tumour cells migrate to the endosteum, tumour cells stimulate the RANKL expression in osteoblasts through the production of PTHrP, prostaglandin E2, interleukin (IL)-6, IL-1β, enhancing osteoclastic bone resorption [48]. Subsequently, osteoclast-induced bone resorption releases calcium. Thus, metastatic tumour cells in the endosteal niche are exposed to high calcium levels. Tumour cells express calcium-sensing receptors (CaSRs) that enable them to detect and respond to extracellular calcium levels, acquiring the ability to migrate and proliferate [52]. Calcium can activate several oncogenic pathways that drive tumour progression. For instance, calcium can influence the expression of genes related to cell cycle regulation, apoptosis and metabolism, further supporting cancer cell growth in bone [53,54]. Besides, the degraded bone matrix induced by osteoclasts also releases TGF-β and IGF, promoting tumour cell proliferation. This interaction among the tumour cells, osteoblasts and osteoclasts develops tumour progression in bone in a process called the ‘vicious cycle.’ In conclusion, the endosteum provides an essential scaffold for bone metastatic tumour cells to grow exponentially and progress into bone-destructing tumours.

## 5. Bone marrow endosteal stem cells in bone tumour initiation

**Figure 3.**
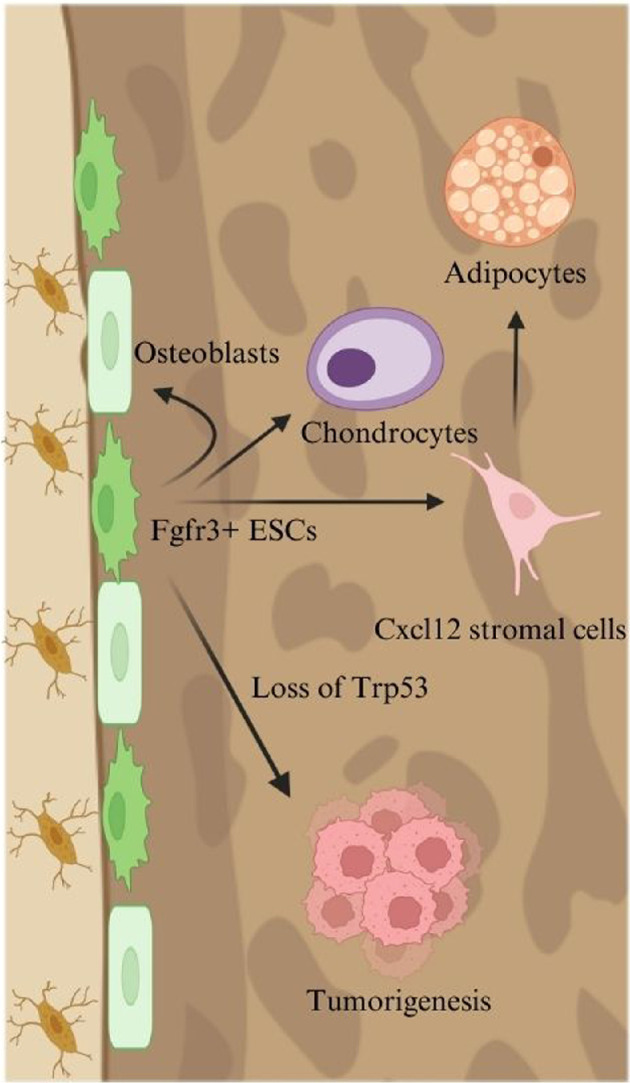
Schematic diagram of the role of Fgfr3^+^ ESCs in bone formation and tumourigenesis. The figure illustrates the differentiation potential and pathological alterations of Fgfr3^+^ ESCs within the bone microenvironment. Under normal conditions, Fgfr3^+^ ESCs contribute to osteoblast formation and are essential for bone maintenance. These stromal cells can also differentiate into Cxcl12-expressing stromal cells, finally differentiating into adipocytes. However, the loss of Trp53 in Fgfr3^+^ ESCs alters their fate, leading to tumourigenesis.

SSCs providing a source of osteoblasts in the endosteum have been poorly characterised [55], with no specific subset of endosteal stem cells (ESCs) identified. Our group has identified that ESCs are characterized by *fibroblast growth factor receptor 3* (*Fgfr3*) expression and osteoblast-chondrocyte transitional (OCT) identities [9] (figure 3). At a young age, these cells have some hallmarks of osteoblasts and chondrocytes, identified by single-cell RNA-seq analysis of bone marrow stromal cells [56]. More specifically, single-cell RNA-seq analyses of *Prrx1-cre-*labelled stromal cells at three weeks (Young) and 18 months (Old) mice (integrated by LIGER [57,58]) reveal three major groups of osteoblasts, chondrocytes and reticular cells. In addition, a small cluster of cells with OCT identities is identified, which express molecular markers of chondrocytes (*Acan*), osteoblasts (*Col1a1*) and reticular cells (*Cxcl12*). These OCT cells are abundant at a young age (P21) but depleted at an old age (18 months), suggesting that OCT cells robustly contribute to active osteogenesis during a young age. Single-nucleus ATAC-seq analyses further confirm that OCT cells maintain multipotent states, indicative of their stem cell potential. *Fgfr3* is a representative marker of these OCT cells, as it is highly expressed in the OCT cluster. Fgfr3^+^ cells in the endosteum have strong colony-forming capabilities *in vitro*, which are often used as proof of stem cell potential.

Osteosarcoma is the most common primary bone tumour, predominantly affecting adolescents and young adults. Osteosarcoma arises from osteoblastic cells that form immature bone or osteoid tissue and primarily occurs in the metaphyseal regions of long bones and the endosteum [59]. The incidence of osteosarcoma is often linked to mutations in tumour suppressor genes and oncogenes [60]. Among these, mutations in the *TRP53* gene (encoding p53) are the most common, occurring in over 50% of cases. The hypothesis is that Fgfr3^+^ ESCs initiate osteosarcoma. We found that the deletion of p53, specifically in Fgfr3^+^ cells with *Trp53*-floxed mice, drives Fgfr3^+^ ESCs to develop into osteosarcoma-like bone tumours, supporting that Fgfr3^+^ endosteal cells can be a cellular source of osteosarcoma (figure 3). Although conventional osteosarcomas most frequently arise in the metaphyseal regions of long bones, several case reports have documented osteosarcomas originating from the cortical bone in paediatric patients [61,62]. Our findings that Fgfr3^+^ ESCs can give rise to osteosarcoma-like tumours during skeletal growth suggest that Fgfr3^+^ ESCs may represent the cellular origin of cortical osteosarcomas in juvenile patients. This insight highlights the potential clinical significance of targeting Fgfr3^+^ ESCs in the context of paediatric osteosarcoma. In conclusion, Fgfr3^+^ cells at the endosteum with SSC-like properties can provide a robust source of osteosarcoma-forming cells.

## 6. Prospects

The bone marrow endosteum is a critical site for haematopoiesis and osteogenesis, hosting a subgroup of hematopoietic progenitor cells and SSCs. Among these, Fgfr3^+^ SSC (‘ESCs’) robustly contribute to the generation of bone-forming osteoblasts, as well as to CXCL12-abundant reticular (CAR) cells and osteocytes within the cortical bone. Fgfr3^+^ cells in the endosteum are heterogeneous, presumably including osteoblast progenitor and precursor cells with varied potential. Moreover, *Fgfr3* is expressed not only in the endosteum but also in the periosteum and growth plate cartilage. As a future endeavour, it will be necessary to identify endosteum-specific SSC markers by combining multiple cell surface markers and isolating endosteal stem cells with high purity for further functional evaluation. This endeavour will be necessary to exploit these cells as potential targets for future innovative therapies.

The bone marrow endosteum provides a supportive microenvironment for the metastatic cancer cells and the progression of haematological malignancies. Most studies have focused on the relationship between tumour cells and drug efficacy. In contrast, relatively few have investigated the interactions between tumour cells and endosteal cells, such as osteoblasts and osteoclasts. As a result, the mechanisms behind bone destruction have not been fully elucidated. This knowledge gap primarily stems from the frequent use of human tumour cells in research, necessitating transplantation into immunodeficient hosts such as nude or SCID mice, limiting the ability to obtain mechanistic insights through genetically modified mouse models.

Recent research has revealed that the periosteum acts as a defensive barrier against cancer invasion into bone [63]. The authors used C57BL/6J-derived head and neck squamous cell carcinoma (HNSCC) cell lines in tumour injection mouse models. They observed that the periosteum highly expresses tissue inhibitor of metalloproteinases 1 (TIMP1). To investigate the role of TIMP1 in the periosteal defence, the researchers used TIMP1 knockout (KO) mice. TIMP1 KO mice exhibited a significant loss of the periosteal defence, allowing cancer cells to infiltrate the bone and proliferate extensively. These findings demonstrate that the periosteum functions as a stromal defence mechanism against cancer invasion. It remains to be determined whether the endosteum functions similarly.

Recent advances in cancer therapies have extended the survival of patients with metastatic bone tumours, MM and AML. However, this has also brought increased attention to the complications of tumor-induced bone destruction, such as bone pain, hypercalcemia, fractures and spinal cord compression, all of which significantly impair patients' quality of life [64,65]. Notably, MM often metastasizes to the calvaria more readily than to long bones such as the femur [66,67]. In some cases, bone destruction facilitates tumour spread into the cranial cavity, leading to extramedullary intracranial growth, which can, in severe cases, result in life-threatening conditions [68]. Therefore, elucidating the crosstalk between tumour cells and the endosteal microenvironment is crucial not only for deepening our understanding of the mechanisms behind cancer-induced bone pathology, but also for developing novel therapeutic strategies to preserve bone integrity and prevent such devastating disease progression.

## Data Availability

This article has no additional data.
